# The Influence of the Urban Environment on Mental Health during the COVID-19 Pandemic: Focus on Air Pollution and Migration—A Narrative Review

**DOI:** 10.3390/ijerph18083920

**Published:** 2021-04-08

**Authors:** Giulia Menculini, Francesco Bernardini, Luigi Attademo, Pierfrancesco Maria Balducci, Tiziana Sciarma, Patrizia Moretti, Alfonso Tortorella

**Affiliations:** 1 Department of Psychiatry, University of Perugia, 06132 Perugia, Italy; giuliamenculini@gmail.com (G.M.); balducci.pierfrancesco@gmail.com (P.M.B.); tiziana.sciarma@unipg.it (T.S.); patrizia.moretti@unipg.it (P.M.); 2 CSM 24 ore Area delle Dolomiti Friulane, Department of Mental Health, AsFO Friuli Occidentale, 33170 Pordenone, Italy; francescobernardini78@yahoo.fr; 3 Planetary Health Lab, Old Medical School, University of Edinburgh, Edinburgh EH8 9AG, UK; luigi.attademo@hotmail.it; 4 SPDC Potenza, Department of Mental Health, ASP Basilicata, Italian National Health Service, 85100 Potenza, Italy; 5 CSM Terni, Department of Mental Health, 05100 Terni, Italy

**Keywords:** COVID-19, SARS-CoV-2, mental health, social determinants, air pollution, climate change, migration, refugees, urban environment, urbanization

## Abstract

The coronavirus disease 2019 (COVID-19) pandemic caused a crisis worldwide, due to both its public health impact and socio-economic consequences. Mental health was consistently affected by the pandemic, with the emergence of newly diagnosed psychiatric disorders and the exacerbation of pre-existing ones. Urban areas were particularly affected by the virus spread. In this review, we analyze how the urban environment may influence mental health during the COVID-19 pandemic, considering two factors that profoundly characterize urbanization: air pollution and migration. Air pollution serves as a possibly risk factor for higher viral spread and infection severity in the context of urban areas and it has also been demonstrated to play a role in the development of serious mental illnesses and their relapses. The urban environment also represents a complex social context where minorities such as migrants may live in poor hygienic conditions and lack access to adequate mental health care. A global rethinking of the urban environment is thus required to reduce the impact of these factors on mental health. This should include actions aimed at reducing air pollution and combating climate change, promoting at the same time a more inclusive society in a sustainable development perspective.

## 1. Introduction

The severe acute respiratory syndrome coronavirus 2 (SARS-CoV-2) infection, resulting in the coronavirus disease 2019 (COVID-19) pandemic, caused a public health emergency not only due to the possible serious consequences of the infection itself, but also in consideration of the subsequent socio-economic crisis [1]. The pandemic also determined a high risk for developing negative emotions among the general population resulting from different factors, such as fear of the contagion, economic burden, and social isolation [2]. In fact, the global situation contributes to creating an environment of disruption and trauma, as it was already demonstrated during previous pandemics [3]. A higher stress perception during pandemics and disasters can thus impair the overall wellbeing of subjects, with effects on sleep, concentration, cognitive function, and behaviors [4]. Specific populations, including subjects affected by COVID-19 or having their relatives affected, health professionals and subjects with pre-existing mental disorders, have been defined as presenting higher risk for developing psychiatric manifestations during the pandemic [5,6,7,8]. The mental health effects of the present pandemic also arose from the implementation of countermeasures such as lockdown and home quarantine. Concerning these, psychiatric manifestations that have been described in the context of lockdown measures mainly belong to the diagnostic categories of depression, anxiety, and psychosomatic disorders [2,9]. In consideration of what stated above and of the high prevalence that mental health consequences of the virus spread demonstrated, mental health needs were pointed out as a priority in the context of the response to the global crisis [10,11]. In addition, SARS-CoV-2 was also demonstrated to exert a direct effect on the central nervous system (CNS), possibly contributing to the occurrence of several neurological manifestations, among which the most frequently reported are hypo/anosmia, dysgeusia or ageusia, dizziness, headache, paresthesia, dysphonia [12]. The neurotropism of COVID-19 may also be directly responsible for behavioral disturbances and mood changes, such as depressive and anxiety symptoms [13].

In this already complex scenario, it is to note that the countermeasures that were established by countries and local governments to control the virus dissemination determined the loss of financial resources for many workers from different sectors [14], seriously affecting employment possibilities [15]. Despite this crisis spread worldwide, the economic consequences of the pandemic are more severe in low-income countries, confirming inequalities among different health systems and resources [16,17]. Furthermore, residents that appear to be more seriously affected by such consequences are the vulnerable ones, frequently presenting scarce economic resources and high rates of unemployment, together with lack of access to health care and poor education about preventive measures [15]. Additionally, urban areas, namely densely populated areas where at least 50% of the inhabitants live in high-density clusters [18], were particularly affected by the virus dissemination, although several factors associated with COVID-19 vulnerability were also reported for rural areas [19,20]. To note, living in urban centers was also found to be associated with the development of psychiatric symptoms among different populations during the pandemic [21,22]. This could be connected with the fact that urbanicity (being born or raised in cities) represents a possible risk factor for the development of serious mental illnesses, such as schizophrenia spectrum disorders and other psychotic disorders [23,24]. Different explanations were sought for this association, including not only social stressors such as inequalities and low social cohesion [25,26], but also possible influences of urbanicity on brain structure [27] and coping styles [28]. The lack of green spaces was also hypothesized to be involved in this relationship, suggesting a higher exposure to pollution and toxins, as confirmed by the evidence that contact with green spaces during the childhood may protect from the later development of psychiatric disorders [29]. 

In the context of the pandemic, urban environment was hypothesized to be extremely affected by the virus dissemination because of higher population density, higher concentration of air pollutants and specific risks associated with lifestyle [21]. Furthermore, the mortality from COVID-19 was significantly higher in metropolitan areas where poorer general health was associated with lower socio-economic and educational status [15]. Among social factors that may significantly influence the response to the pandemic in urban areas, the presence of ethnic minorities and higher international migration rates were listed among relevant factors, possibly being connected with inequalities in access to health care and relevant socio-economic load [19]. Migration itself is as well connected to mental health problems, since it has been demonstrated that different phases of the migration process may cause a higher risk for developing serious psychiatric disorders, particularly psychosis [30]. The above-mentioned social determinants also influence the emergence of mental health problems, acting as mediators of the COVID-19 psychological impact [31]. Furthermore, social determinants themselves are also expected to be seriously influenced by the pandemic [32], with the risk of implementing a vicious cycle possible leading to even more severe consequences of the present situation on mental health. 

In this narrative review, we analyze the role of urban environment as a possible mediator of the effect that COVID-19 may exert on mental health. In particular, the present review will be focused on air pollution and migration. These two apparently different factors were chosen in consideration of the significant association that they already demonstrated with mental health, as both were considered to present a causal relationship with psychiatric disorders. Furthermore, air pollution and urbanicity are among the factors that most represent the complexity of urban environment under different perspectives. Indeed, the first is connected with anthropogenic activities that are typical of the urban environment, i.e., industries, whilst the second profoundly affects the social texture of urban societies and mirrors social inequalities in this context. We hypothesized that the analyzed literature may support a stronger association between COVID-19 and mental health in urban contexts as possibly mediated by these two phenomena, confirming their importance as social and environmental determinants of mental health. 

## 2. Methods

A comprehensive literature search was performed in the research databases PubMed/Index Medicus/MEDLINE, Scopus and Web of Science, by variously combining the words “COVID-19”, “SARS-CoV-2”, “mental health”, “urban*”, “air pollution”, “pollutant*”, “migrant*”, “migration”, “refugee*”, “asylum seeker*” until 31 December 2020. We included papers in English, French, Italian and Spanish that reported data concerning the impact of the pandemic on mental health with particular attention to the considered determinants related to the urban environment, namely air pollution and migration. In consideration of the relatively recent emergence of the pandemic, papers that considered the possible role of the above-mentioned factors in the occurrence and spread of the pandemic itself were also included. Furthermore, due to the possible communication of preliminary data concerning the considered aspects, we did not limit to the inclusion of full-length original articles, but also included reviews, commentaries and letters to the editor. We excluded papers that did not provide sufficient information concerning possible causal relationships between air pollution and related phenomena, mental health, and COVID-19, as well as papers providing only a theory or hypothesis not supported by sufficient data.

## 3. Results

The literature search yielded 663 records (226 PubMed/Index Medicus/MEDLINE, 278 Scopus, 159 Web of Science). After the whole screening process, including hand-screening of references, was completed, 33 papers were included in this review. Among these, 14 focused on air pollution and related phenomena, whilst 19 focused on migration. For a list of the included papers concerning the two main topics of this review see Appendix A. 

### 3.1. Urban Environment and Mental Health: The Role of Air Pollution

Air pollution has already been listed among the factors associated with higher viral transmission and COVID-19 severity. This could be due to the proven role of atmospheric particulate matter (PM) in creating an environment where the virus survival is facilitated for hours, causing the widespread via airflows over large distances [33]. Additionally, air pollutants such as nitrogen dioxide and carbon dioxide can contribute to the development of a serious inflammatory response that mainly concerns the respiratory system, which represents a possible reason for the higher severity of COVID-19 observed in highly polluted regions in China and Northern Italy [34,35]. Noteworthy, high levels of air pollution could also act synergistically with the virus in its already mentioned neurotrophic mechanism [15]. Furthermore, air pollutants contribute to the phenomenon of global warming, which seriously affects climate change. Modifications in meteorological parameters, such as temperature, are also connected with climate change and were demonstrated to facilitate the infection spread. Indeed, both higher and lower temperatures appeared to be beneficial in decreasing COVID-19 transmission, whilst average temperatures were linked to higher possibility of viral spread [36,37]. Nanoparticles that contribute to air pollution can reach the CNS via the olfactory-neural tract, activating several pathophysiological pathways that include cerebrovascular dysfunction, oxidative stress, inflammatory processes, activation of the immune system, damage to blood vessels, alterations in neurotransmitter concentrations, and alterations in the blood-brain barrier [38,39], possibly playing a role in the pathophysiology of neuropsychiatric symptoms that are strictly connected with the infection. Recently increasing literature is investigating negative impacts of air pollution exposure on mental health. In particular, air pollutants exposure seems to be associated with a higher risk of severe mental disorders [40,41,42], as well as with a higher number of hospital admissions for psychiatric reasons [43,44]. Further phenomena that were linked to air pollution are represented by suicidal ideation and suicidal behaviors [45,46,47]. To note, urbanicity has been commonly described as one of the risk factors for the onset of schizophrenia spectrum disorders, and air pollution exposure could be considered as a potential mediator of the association between urbanicity and the risk of both psychotic disorders and viral epidemics or pandemics [48,49,50]. Moreover, air pollution indirectly affects mental health by causing climate change, that can be responsible for natural disasters and extreme events, leading to mental distress and to psychiatric disorders such as post-traumatic stress disorder (PTSD) [51,52]. Furthermore, gradual climate changes could play and indirect role in the emergence of psychiatric symptoms, i.e., contributing to social changes and migration phenomena, but also inducing negative emotional responses such as anxiety and sense of guilt for the ongoing situation [53]. The association of both air pollution and COVID-19 with mental health problems led to the hypothesis that these phenomena may to some extent be all linked [48].

It has also been demonstrated that urbanicity could represent a possible risk factor for the spillover phenomenon, facilitating the virus transition from animals to humans via intermediate hosts. Particularly, deforestation policies may facilitate this process, resulting in the destruction of natural habitats of numerous species and reduction of biodiversity, as well as in greater interaction between wildlife and human activity [54]. At the same time, during the COVID-19 pandemic, deforestation registered an increasing trend, probably due to socio-economic reasons that were exacerbated by the global situation [55]. This is expected to facilitate the interaction between humans and wild animals, leading to a vicious circle that may cause the emerge of new diseases. Furthermore, airborne particles may result from forest fires, increasing pollutants level in surrounding areas, which suggests that the current situation could also potentially increase the burden of pollution-related medical conditions [33,56].

Anyway, the relationship between air pollution and COVID-19 presents a multi-facet nature. In fact, the countermeasures adopted for containing the viral spread, such as social distancing and home quarantine, led to several environmental changes, above all the reduction of toxic emissions produced by industries and other anthropogenic activities [17,57]. On the other side, higher levels of household air pollution might be associated with quarantine measures since indoor anthropological activities significantly increased during this timeframe [58]. Noteworthy, depressive symptoms were associated with living in small apartments characterized by poor housing, such as scarce air and lighting quality [59]. Higher risk for developing depression in the middle-aged and older population was found to be associated with indoor air pollution caused by solid fuels (i.e., coal) when compared to “clean” sustainable fuels (i.e., natural gas) [60]. Above all, being subjected to lockdown measures was demonstrated to facilitate the emergence of psychiatric symptoms, that significantly increased with the persistence of such measures [11]. To this end, future studies are expected to clarify to which extent these symptoms may be mediated by indoor pollution. Additionally, the presence of green spaces in living environments demonstrated a link with a reduction of perceived stress. This could be in part confirmed by the evidence that interventions based on video-audio stimuli reduced anxiety levels in subjects undergoing lockdown and quarantine measures, with a higher efficacy when forest environments were displayed [61]. In addition, the access to green spaces was reported as a crucial need during the pandemic, also connected with the possibility for physical exercise and relaxation. At this regard, citizens who underwent quarantine measures also suggested to improve urban plans to project wider big areas in metropolitan centers, which was hypothesized to determine an improved quality of life [62].

The sensibilization about green policies could also allow a better perception of actions required for contrasting climate change [63]. This represents a relevant issue, especially in countries where climate-induced natural disasters frequently occur, since food deficiency and lack of medical assistance during such events is expected to be even more critical in consideration of the ongoing pandemic situation [14]. Indeed, climate-related calamities, i.e., heat waves, hurricanes, cyclones, still represent a global threat for under-resourced health systems, that may not be able to guarantee an adequate response [64]. This could particularly affect overloaded mental health professionals, which would be conversely expected to implement different actions in order to face these disasters, providing psychosocial treatments and improving technology-based interventions that could be spread on large scale [65].

### 3.2. Urban Environment and Mental Health: Focus on Migrant Populations Mental Health during the COVID-19 Pandemic

The public health needs of minorities such as migrants generally represent a relevant issue in modern societies, which should be afforded by means of culturally competent services. As stated above, the pandemic situation represents a factor which may potentially increase the vulnerability of this population, since anxiety related to COVID-19 could overlap with worries concerning the precariousness of their condition and the lack of a regular working status [66].

In this context, undocumented migrants, asylum seekers, refugees and those living in camps and detention centers may be subjected to a dramatic situation due to difficulties in adhering to public health directives and to specific environmental conditions that may result in higher risk for contracting the infection [67]. As for refugee camps, these were particularly affected by the viral spread, as demonstrated by specific cases of COVID-19 outbreaks, i.e., in European countries such as Greece [68] and Malta [69]. In these settings both direct and indirect pathways of transmission should be considered. Indeed, overcrowding is frequent and may hinder the implementation of social distancing measures [70]. At the same time, adequate facilities for hygiene measures such as handwashing may not always be available. Additionally, in settings with a heavy viral contamination, the contagion could spread by fingertip contact with infected surfaces and it has also been hypothesized that the transmission could be mediated by food [71].

Refugee camps do not represent the only cause due to which these populations may be exposed to high infectious risk. For example, asylum seekers whose request of international protection has been rejected do not own regular documents and are most frequently homeless, living in conditions of poverty due to lack of work [1]. In such cases, the COVID-19 spread could also cause changes in the humanitarian corridors and asylum seekers may be returned to their countries of origins, where they are at risk of being persecuted [72]. For similar reasons, in some countries asylum seekers and undocumented migrants can avoid seeking help for health matters due to fear of being repatriated [73,74]. Rescue operations in the Mediterranean Sea were suspended as well due to logistic reasons, whilst the few that were carried led to quarantine measures in refugee camps with subsequent organizational concerns [72]. Furthermore, shortage of food and medicines that already affected refugee camps in several parts of the world could be exacerbated during the pandemic, adding further concerns to administrative, socio-economic, legal and language barriers in accessing health care [72,75]. These conditions can foster feelings of uncertainty and loneliness that often prelude to the onset of anxiety and depressive symptoms. Additionally, pre-existing mental disorders that are particularly prevalent among this population may be exacerbated during the pandemic, also representing an obstacle to the recognition of specific symptoms and determining a higher risk for the infection spread [67].

Indeed, migrants and especially asylum seekers represent a vulnerable group of individuals, facing traumatic events during different phases of the migration process, i.e., childhood abuse, armed violence, detention, and isolation [76], often leading to the development of PTSD, adjustment disorders and depressive symptoms [1]. Such symptoms may also be influenced by difficulties in adaptation to the culture of the host country, poverty, and racism [77]. The pandemic can act as a trigger for the recall of traumatic experiences, as demonstrated by a study conducted in a refugee camp in Iraq where different PTSD symptoms measured by the Impact of Event Scale-Revised (IES-R) were more severe after the COVID-19 outbreak than they were before [78]. Not only did fear of illness and death, as well as concerns for safety of the loved ones cause symptom exacerbation, but also social distancing might have contributed to more serious psychopathology by hindering the creation of social networks that could help refugees to connect with the host culture and society [79]. This could further be exacerbated by quarantine measure, which cause anger and confusion and could evoke memories of restricted freedom to those who experienced imprisonment in their past. Moreover, refugees often escaped oppressive regimens during and can perceive the reinforcement of police and military presence to help respecting restrictions as a threat to personal security [79]. Symptom exacerbation during the pandemic, together with limited access to mental health care and lack of adequate psychological support, can lead to serious consequences and to a higher suicide risk [80]. Results from a population of individuals in low socio-economic conditions, including migrants, detected a significantly lower percentage of users after the adoption of lockdown measures, with fewer subjects attending follow-up visits during the next months [1], demonstrating the need for a reorganization of mental health services that are addressed to this population. Tailored psychological aid programs for refugees were implemented, based on informative materials about the virus spread and on mental health condition monitoring by means of phone and, when possible, using telemedicine [81]. This latter resource, despite limitations due to scarce internet access and setting variation, allowed in some cases to continue treatment programs especially in young subjects [81].

Another population that may suffer from mental health problems related to the CoV-Sars-2 spread is represented by international migrant workers [82], 95% of which are living in regions affected by the pandemic [83]. Migrant workers, especially in urban areas, often engage in occupations characterized by low wages and experiment a condition of global uncertainty, facing social and cultural barriers [84]. Furthermore, these individuals are prone to the development of psychiatric disorders [85,86,87] and are frequently affected by comorbid medical illnesses, also due to poor hygienic conditions and chronic malnutrition [82]. Factors that contribute to the development of mental health problems in this population are loneliness, lack of familiar support [88], social exclusion [89], and difficulties in accessing psychiatric care [82]. Due to the pandemic situation, these individuals are expected to cope with serious economic load due to job loss in the next future, as already demonstrated in some areas of the world [87], and could be subject to an inverse migration phenomenon, thus returning to their native villages [90], which was also demonstrated to be a risk factor for suicide [72]. Language barriers create further limitations to the acquisition of adequate information about the public health situation and personal protection [83], with the latter becoming even more difficult to address for migrant workers living in shelter and camps [84]. These issues, together with perceived and internalized stigma and low education level, hinder the access of this population to specific psychological aid services that were settled in some countries during the pandemic [87,91,92,93]. To note, the stigmatization of minorities underpins a process through a specific human characteristic is labeled as socially salient and is usually considered under a discrediting point of view [94]. This phenomenon, that has historically been associated with psychiatric illness in modern societies, gains further relevance when considering mental health among minorities.

We should also consider that international migrant workers are also affected by worries for their families of origin living in countries that are highly affected by the COVID-19 pandemic [95], expressing their struggle to travel to native countries and meet the loved ones, despite adjunctive quarantine measures that they usually have to undergo [84,87]. This could further facilitate the virus transmission towards native villages and countries which could initially be less affected by the pandemic, since migrations was listed among the main reasons for the long-distance viral spread [96].

In a cross-sectional study based on self-reports and interviews, migrant workers reported a high rate of negative emotions including frustration, fear, and irritability [96]. In the same population, 75% of migrants screened positive for anxiety or depression, with the first being more frequent than the latter [97]. In a research focusing on Italian foreign workers, PTSD was detected among about 22% of them and was significantly predicted by the development of anxiety and depression [95]. Another study where migrant workers living in camps and shelters were interviewed, a high burden of substance abuse emerged, determining further issues during the pandemic due to withdrawal symptoms and craving for substances that could not be easily accessed [84].

## 4. Conclusions

In this narrative review, we underlined how different aspects that characterize the urban environment can contribute to the SARS-CoV-2 spread and increase the COVID-19 pandemic complexity. Indeed, the current situation seriously affects public health, but it also presents relevant implications under a socio-economic point of view. The overall severity of the emergency was demonstrated to present relevant implications for mental health as well. Noteworthy, several factors connected with the urbanization process are associated with such implications. Among these, air pollution represents a potential link between COVID-19 and mental health, since it was proved to be a risk factor for the development of both conditions. Furthermore, this phenomenon is also connected with major issues that have relevant societal consequences and particularly, but not only, climate change. Additionally, the urban environment presents a social structure that often leads to inequalities and hampers the access to adequate health care for specific populations.

**Figure 1:**
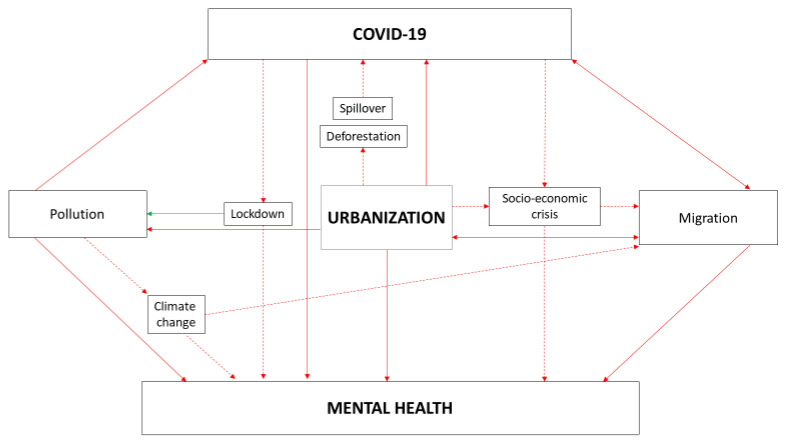
Possible causal relationships between COVID-19, mental health and urbanization.

This was particularly demonstrated for minorities such as refugees, asylum seekers and migrant workers, that can be subjected to major issues concerning the maintenance of adequate hygienic conditions, determining a higher risk of contagion in the pandemic era. Additionally, during the pandemic access to health facilities may be even more difficult, with relevant influence on general health and particularly on mental health. Several factors connected with the migrant condition, as well as the high prevalence of pre-existing psychiatric disorders, contribute to the significant burden that mental health issues cause in this population during the COVID-19 pandemic. Possible causal relationships between COVID-19, mental health, and urbanization are described in Figure 1.

Although we considered only some among the possible determinants of mental health, the evidence we summarized suggests the need for a comprehensive rethinking of the urban environment. This represents a crucial topic since fifty per cent of the world population lives in densely populated urban areas and further urbanization is expected during the next decades. The promotion of human behaviors aimed at reducing air pollution and contrasting climate change, together with a more sustainable exploitation of natural resources in populated areas, represent unique possibilities to improve human health, with significant influence on mental health as well. Specific initiatives focused on the maintenance of biodiversity, the improvement of urban green cover and the promotion of agriculture activities in adjacent areas may help reaching these goals. Greater attention should also be dedicated to indoor environments, as suggested by some of the reported literature.

Noteworthily, the COVID-19 pandemic led to a global change concerning the possibility for long-distance working, as well as to a redefinition of activities that may be held in the context of households. Furthermore, the reduction of inequalities that could be exacerbated in urban environments should also be considered as a priority. Migration phenomena are not expected to decrease in the next decades, and most of the global population will be living in urban areas. In this context, policies aimed at social and economic inclusion could significantly affect the burden of mental health problems related with a distressing marginalization condition. The promotion of access to health care, with particular attention to mental health care, should be strongly implemented among minorities. Noteworthy, mental health and well-being were also included in the United Nations Sustainable Development Goals, together with the implementation of sustainable cities and communities, inequalities reduction and climate change-related issues, suggesting that these are all aspects of a multi-facet global change program. In order to address these goals, a multidisciplinary approach is required, involving the participations of professionals working in public health and specialists in different medical fields, as well as experts in sociology, engineering, architecture, and environmental sciences.

Despite the lack of a systematic approach that might have led to the inclusion of heterogeneous literature, i.e., not defining urban areas clearly, the present review presents among its strength the consideration of mental health under a comprehensive approach, taking into account general health and socio-economic determinants. Future prospective studies targeting mental health in urban areas in the COVID-19 era may better address the issues raised in this paper.

In conclusion, the COVID-19 pandemic and the related mental health problems raise several issues that lay the foundations for future strategies oriented towards a more sustainable and inclusive planning of urban environments. This also depends on higher awareness concerning social determinants of mental health, as well as an overall reduction of social stigma that affects both mental health and minorities.

## Appendix A

**Table A1:** Papers evaluating the relationship between air pollution and related phenomena, the COVID-19 pandemic and mental health.

Author (Year) [Reference]	Article Type	Study Design (for Studies with Human Sample)
Bodrud-Doza et al. (2020) [14] (pp. 2, 6)	Original research	Cross-sectional survey
Chakraborty et al. (2020) [17] (pp. 2, 5)	Review	Not applicable
Wu et al. (2020) [34] (p. 4)	Original research	Cross-sectional study
Conticini et al. (2020) [35] (p. 4)	Commentary	Not applicable
Tosepu et al. (2020) [36] (p. 5)	Original research	Retrospective study
Li et al. (2021) [37] (p. 4)	Original research	Not applicable (no human sample)
Marazziti et al. (2021) [48] (p. 5)	Review	Not applicable
Brancalion et al. (2020) [55] (p. 5)	Policy Forum	Not applicable
Venter et al. (2020) [57] (p. 5)	Original study	Not applicable (no human sample)
Sharma et al. (2020) [58] (p. 5)	Letter to the editor	Not applicable
Amerio et al. (2020) [59] (p. 6)	Original research	Cross-sectional survey
Zabini et al. (2020) [61] (p. 6)	Original research	Interventional study
Ugolini et al. (2020) [62] (p. 6)	Original research	Cross-sectional survey
Barouki et al. (2020) [64] (p. 6)	Review	Not applicable

**Table A2:** Papers evaluating the relationship between migration, the COVID-19 pandemic and mental health.

Author (Year) [Reference]	Article Type	Study Design (for Studies with Human Sample)
Aragona et al. (2020) [1] (pp. 1, 6, 7)	Research article	Naturalistic study
Liu et al. (2020) [60] (p. 5)	Commentary	Not applicable
Bhopal et al. (2020) [66] (p. 6)	Letter to the editor	Not applicable
Ralli et al. (2020) [67] (pp. 6, 7)	Review	Not applicable
Kluge et al. (2020) [72] (pp. 6, 7)	Letter to the editor	Not applicable
Mukumbang et al. (2020) [73] (p. 6)	Review	Not applicable
Page et al. (2020) [74] (p. 6)	Commentary	Not applicable
Dalexis and Cenat (2020) [76] (p. 7)	Letter to the editor	Not applicable
Kizilhan and Noll-Hussong [78] (2020) (p. 7)	Letter to the editor	Naturalistic study
Rees et al. (2020) [79] (p. 7)	Review	Not applicable
Endale et al. (2020) [81] (p. 7)	Experiential account	Experiential account
Choudhari (2020) [82] (p. 7)	Review	Not applicable
Liem et al. (2020) [83] (p. 7)	Letter to the editor	Not applicable
Chander et al. (2020) [84] (pp. 7, 8)	Experiential account	Experiential account
Espinel et al. (2020) [90] (p. 7)	Commentary	Not applicable
Chan and Kuan (2020) [93] (p. 7)	Research article	Naturalistic study
Barbato and Thomas (2020) [95] (pp. 7, 8)	Letter to the editor	Cross-sectional survey
Fan et al. (2020) [96] (p. 8)	Research article	Retrospective study
Kumar et al. (2020) [97] (p. 8)	Letter to the editor	Cross-sectional survey
